# Non-enveloped virus inactivation potency of sodium dodecyl sulfate with citric and glutamic acids

**DOI:** 10.3389/fmicb.2025.1535404

**Published:** 2025-02-19

**Authors:** Yong Wah Tan, Chwee Fern Bok, Brenda Jun Fang Lim, Li Ying Kong, Kian Sim Goo, Yoshiki Ishida, Jiquan Liu, Chun Song Chua, Justin Jang Hann Chu

**Affiliations:** ^1^Institute of Molecular and Cell Biology (IMCB), Agency for Science, Technology and Research (A*STAR), Singapore, Singapore; ^2^Singapore Innovation Center, The Procter & Gamble Company, Singapore, Singapore; ^3^Biosafety Level 3 Core Facility, Yong Loo Lin School of Medicine, National University of Singapore, Singapore, Singapore; ^4^Laboratory of Molecular RNA Virology and Antiviral Strategies, Department of Microbiology and Immunology, Yong Loo Lin School of Medicine, National University of Singapore, Singapore, Singapore; ^5^Infectious Disease Translation Research Programme, Yong Loo Lin School of Medicine, National University of Singapore, Singapore, Singapore

**Keywords:** disinfectant, virus inactivation, non-enveloped virus, sodium dodecyl sulfate, citric acid, glutamic acid

## Abstract

Disinfection is one of the most important methods by which transmission of infectious diseases can be blocked, and efficacies differ depending on how they are used and the target organism. Small non-enveloped viruses are considerably less sensitive to disinfectants than enveloped viruses and vegetative bacteria or fungi and generally require strong protein-disrupting chemicals for effective inactivation, limiting their application in personal care products due to associated side effects. Sodium dodecyl sulfate (SDS) is a common anionic surfactant and relatively safe ingredient used in many personal care and hygiene products possessing protein-denaturing properties and has been reported to have antimicrobial efficacy against enveloped viruses and bacteria. With the aim of identifying milder disinfectants with broad-spectrum activity, including efficacy against non-enveloped viruses that are more difficult to inactivate, this study focused on evaluating the combinatorial efficacy of sodium dodecyl sulfate with organic acid (i.e., citric acid) and amino acid (i.e., glutamic acid) on feline calicivirus. Using an *in vitro* quantitative suspension test and electron microscopy, we have demonstrated the virucidal efficacy of SDS combinations with citric or glutamic acids on FCV. In addition, the spectrum of virucidal efficacy may potentially be extended to some human enteroviruses, and further research into their variable sensitivity to virus inactivation would be useful in developing these combinations into consumer products that target non-enveloped viruses.

## Introduction

Highlighted by the SARS coronavirus 2 pandemic, viruses continue to be a burden on both health and the economy. While efforts on vaccine and antiviral development against a myriad of infectious viruses and government intervention are important strategies in controlling the transmission of infectious diseases, especially during outbreaks, practicing proper personal hygiene plays an important role in preventing the spread at the individual level.

The susceptibility of viruses to disinfection agents varies broadly, with the small non-enveloped viruses being the most resistant to inactivation and generally requiring chemicals that disrupt protein structures such as sodium hypochlorite and glutaraldehyde (Lin et al., 2020). Both sodium hypochlorite and glutaraldehyde can cause skin irritation, limiting their safety and efficiency in personal care products. While some products contain sodium hypochlorite, they are used in the treatment of conditions such as atopic dermatitis (Lee and Van Bever, 2014).

Sodium dodecyl sulfate (SDS) is a common additive in personal care products and an anionic surfactant with protein denaturation potency. These chemical properties are believed to be crucial to virus inactivation efficacy by binding to and disrupting the lipid bilayer of enveloped viruses, as well as disabling or denaturing viral capsid proteins (Simon et al., 2021). SDS has been shown to inactivate papillomaviruses (Howett et al., 1999), and combinations of SDS, particularly with acids as SDS is known to function at low pH, have been tested for antimicrobial properties, including inactivation of selected viruses assessed using different methods (Aydin et al., 2013; Zhou et al., 2017; Maktabi et al., 2018).

Feline calicivirus (FCV), a non-enveloped virus, is a widely used surrogate for human noroviruses (EPA US, 2023), the main viral etiology of acute gastroenteritis worldwide. This is due to the lack of *in vitro* culture platforms for human noroviruses, which have been well-established for FCV. Using FCV as a model organism, we have explored the virus inactivation potencies of SDS combinations with several organic acids and amino acids and found that combinations containing either citric acid (CA) or glutamic acid (GA) exhibited good efficacy (data unpublished) based on suspension tests performed at room temperature (≈ 25°C) at pH 2.5. Nevertheless, the low pH requirements for virucidal efficacy would limit their application in consumer and hygiene products due to potential safety issues associated with low pH. Hence, in this study, we determined and optimized the efficacy of the two combinations at higher pH using an *in vitro* suspension virus inactivation assay and assessed the potential spectrum of activity against other viruses.

## Materials and methods

### Viruses and cell lines

Feline calicivirus (FCV) (strain F-9, VR-782), vaccinia virus (VACV) (strain Elstree, VR-1549), human rhinovirus (HRV) 39 (strain 209, VR-340), influenza A virus (FLUAV H1N1) (strain A/PR/8/34, VR-1469), and murine norovirus 1 (MNV-1) (strain CW1, VR-1937) were purchased from ATCC. Human enterovirus A71 (EV-A71) (strain 5865/SIN/000009), D68 (EV-D68) coxsackievirus A6 (CV-A6), CV-B5, echovirus 7 (E-7) (strain Wallace), chikungunya virus (CHIKV), dengue virus type 2 (DENV-2), infectious bronchitis virus (IBV), murine hepatitis virus (MHV), and severe acute respiratory coronavirus 2 (SARS CoV-2) were laboratory isolates. Cell line and culture media information are summarized in Supplementary Table S1.

### Preparation of virus stock and test samples

Non-enveloped viruses were harvested from infected cells using one freeze/thaw cycle. The culture media containing cell lysate was cleared by filtration using a 0.2-μm polyethersulfone (PES) filter unit and then stored in the −80°C freezer as single-use aliquots. Enveloped viruses were harvested using the same process without freezing/thawing. Due to the nature of some viruses, especially enveloped viruses, the virus titers could not be standardized to 1 × 10^7^ PFU/mL for all virus stocks. Viral plaque assays were performed for each pool and determined to be >1 × 10^7^ PFU/mL (FCV, EV-A71, CV-A6, CV-B5, E-7, HRV-39, MNV-1, and FLUAV), >1 × 10^6^ PFU/mL (VACV, DENV-2, CHIKV, MHV, IBV, and EV-D68), and > 1 × 10^5^ PFU/mL (SARS CoV-2).

For the preparation of test samples, all ingredients were diluted in ultrapure water, and pH was adjusted using hydrochloric acid and sodium hydroxide, followed by filtration through a 0.2-μm surfactant-free cellulose acetate syringe filter before use. All test samples were used within 24 h to minimize pH shifts.

### Virucidal efficacy

The BS EN 14476:2013 + A2:2019 quantitative suspension test under clean conditions was used to evaluate the virucidal efficacy of test samples. *Eight* parts of the test sample were mixed with one part of 0.3% BSA and one part of the virus and then incubated at room temperature for 10 min or otherwise stated. A measure of 100 μL of the reaction mixture was then diluted with 900 μL of neutralizer before the samples were 10-fold serially diluted with media for the viral plaque assay. The neutralizer used for all experiments was Modified Letheen Broth (DIFCO) supplemented with Tween-80 and lecithin to 1.5 and 1%, respectively.

### Viral plaque assay

For FCV, monolayers of Crandell-Rees Feline Kidney (CRFK) cells in 24-well plates were inoculated with 100 μL of serially diluted reaction mixtures and incubated at 35°C for 1 h (h). The inoculum was then removed and overlaid with media containing 0.8% carboxymethyl cellulose (CMC) (Sigma-Aldrich). The inoculum was returned to the incubator for 48 h then fixed and stained in 4% paraformaldehyde with 0.25% crystal violet to visualize viral plaques. For other viruses, the overlay media and culture temperatures are summarized in Supplementary Table S2. For all plaque assays, virus titers were obtained as an average of three biological replicates, with three technical replicates per biological set.

### Negative stain transmission electron microscopy

One part of the virus suspension (4 × 10^8^ PFU/mL) was treated with four parts of the test sample, incubated at room temperature for 10 min, and fixed in 2.5% glutaraldehyde (Sigma-Aldrich). After fixation, the virus was adsorbed onto formvar/carbon film-coated copper grids (Electron Microscopy Sciences), followed by staining with 1% phosphotungstic acid (PTA). Grids were air-dried before viewing with the transmission electron microscope JEM-1400 (JEOL Ltd.). Images presented are representative of minimally six coated grids imaged on three separate occasions.

## Results

As prior experiments (unpublished) were conducted at a low pH of 2.5 and at rather high concentrations of SDS and CA or GA, we first tested the virus inactivation efficacy of the two combinations at different pH values to find a combination that can work at a pH closer to neutral to make it more suitable for close contact with human skin. The results showed that at the high concentration of 5% SDS and 1% CA or GA, FCV can be effectively inactivated within 10 min at both pH 2.5 and 4.7 (Table 1). Since both combinations were able to inactivate FCV at pH 4.7, subsequent studies at lower ingredient concentrations were performed at pH 4.7.

**Table 1:** FCV titer reduction after treatment with SDS, SDS + CA, or SDS + GA combinations at different pH values.

SDS (%)	Citric acid (CA) (%)	Glutamic acid (GA) (%)	pH	Log_10_ titer reduction	Maximum reduction (*)
5			2.7	3.423	*
5	1			3.423	*
5		1		3.423	*
5			4.7	0.038	
5	1			3.423	*
5		1		3.423	*
5			7	0.221	
5	1			0.133	
5		1		0.093	
5			10	0.156	
5	1			0.158	
5		1		0.282	

Titer reduction was calculated using FCV treated with sterile distilled water (pH 7) as a control. Virus titer is below the detection limit (maximum reduction) when no plaques were observed at the lowest non-cytotoxic concentration of each sample in viral plaque assays (Supplementary Table S3).

**Figure 1:**
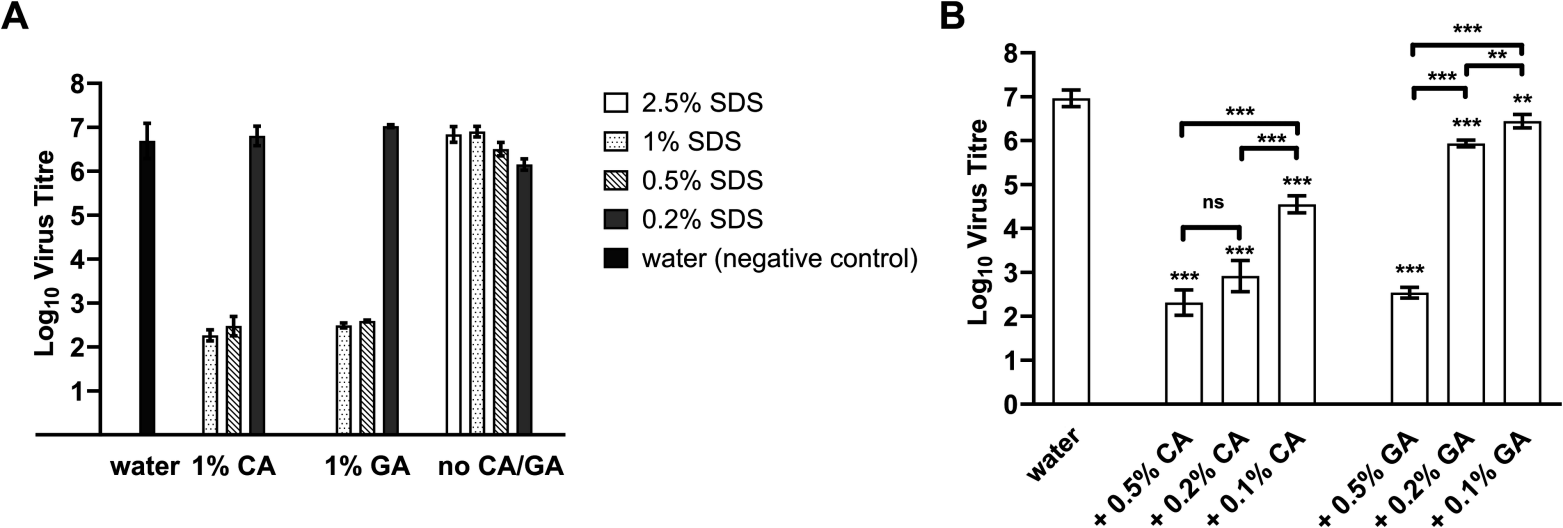
FCV titer after treatment with SDS + CA and SDS + GA combinations at different concentrations. **(A)** Concentration of SDS was lowered from 2.5 to 0.2% in combination with 1% CA, 1% GA, or in the absence of either acid (no CA/GA). A water sample was included as a negative control. No virus was detected for FCV treated with samples of 2.5% SDS + 1% CA and 2.5% SDS + 1% GA. Statistical analysis using one-way ANOVA was performed using Tukey’s multiple comparison test and presented in Supplementary Table S5. **(B)** FCV was treated with samples containing 0.5% SDS + CA or GA at different concentrations. A water sample was included as a negative control. One-way ANOVA was performed using Tukey’s multiple comparison test to compare virus titers of FCV between different sample treatments (ns: not significant, **: *p* < 0.01, *** *p* < 0.001).

The concentration of SDS was then lowered in the combinations, and it was found that both were still highly effective at 2.5% SDS (Figure 1A) as the FCV titer was reduced below the detection limit (3 log_10_ units); however, the combinations were also more cytotoxic (Supplementary Table S4) than combinations with lower concentrations of SDS. Since 1 and 0.5% SDS were found to possess similar virus inactivation efficacy in combination with both acids, the concentration of SDS was fixed at 0.5% to determine virus inactivation efficacies with CA or GA at lower concentrations of 0.5, 0.2, and 0.1%.

A concentration-dependent change in virus inactivation efficiencies of the combinations was observed for both SDS + CA and SDS + GA combinations when the acid concentration was reduced (Figure 1B) with SDS kept at 0.5%. Specifically, reducing the CA concentration of SDS + CA combinations to 0.5 and 0.2% resulted in the incomplete inactivation of FCV samples; residual viable virus was detected close to the detection limit, although the reduction of virus titer was more than 4 log_10_ units. For SDS + GA combinations, at 0.5% GA, virus inactivation efficiency was similar to CA, and a reduction of virus titer by more than 4 log_10_ units was observed. At 0.2 and 0.1% GA, virus inactivation efficiency was severely compromised with a reduction of approximately 1 and 0.5 log_10_ units, respectively. The optimal concentrations for both combinations have been determined to be 0.5% SDS + 0.5% CA and 0.5% SDS + 0.5% GA, respectively, for the lowest cytotoxicity and highest efficacy, with a contact time of 10 min.

**Figure 2:**
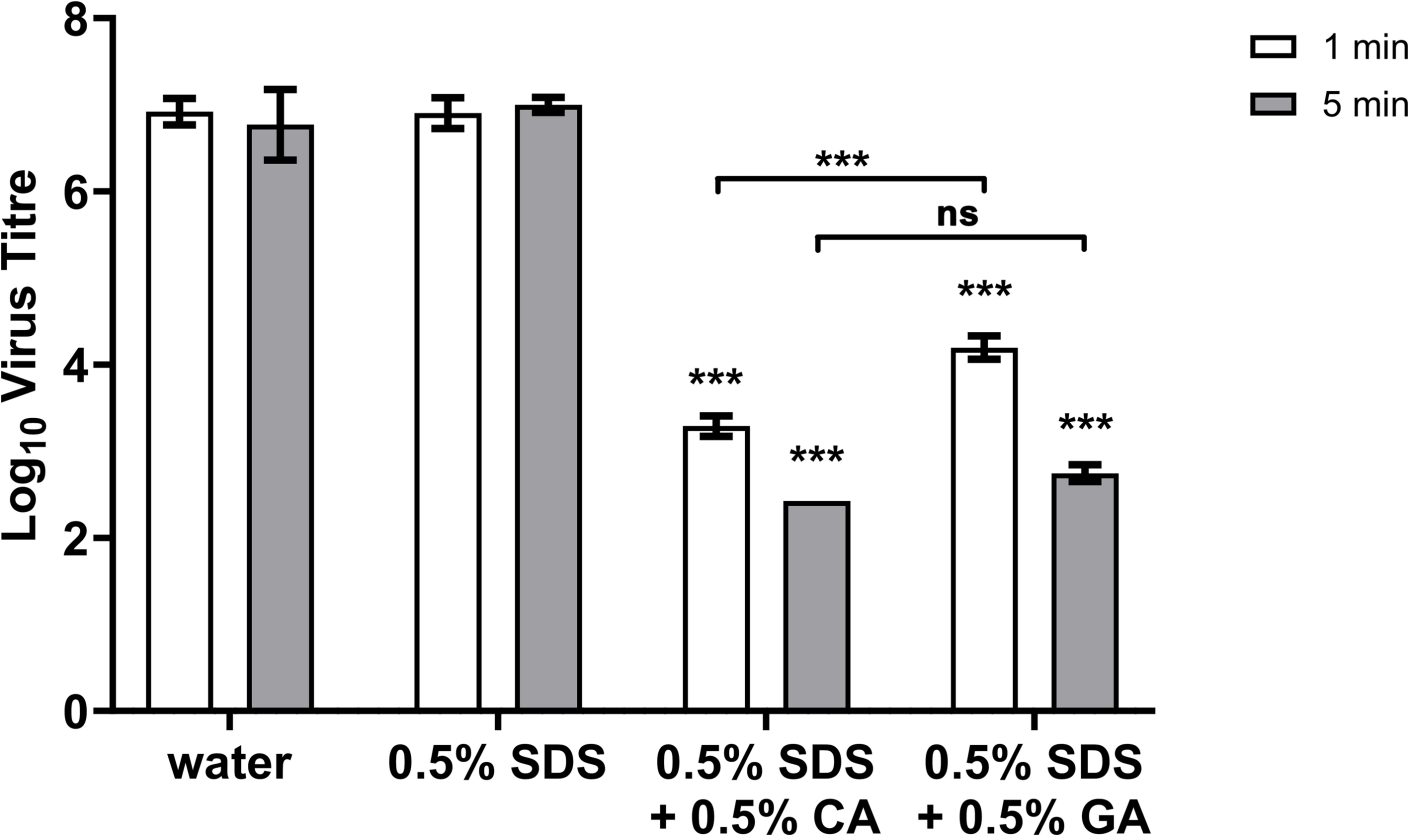
FCV titer after 1- or 5-min treatment with 0.5% SDS + 0.5% CA or GA. FCV was incubated with indicated combinations of SDS, CA, and GA for 1 or 5 min. Statistical analysis was performed using one-way ANOVA with Dunnett’s multiple comparison test against negative control (water) for each contact time, ***: *p* > 0.001; no significant difference was noted for 0.5% SDS-treated FCV samples.

We looked into the speed of virus inactivation and found both 0.5% SDS + 0.5% CA and 0.5% SDS + 0.5% GA combinations to be similarly effective after 5-min contact time (Figure 2) reducing the virus titers by ≈ 4.3 and 4 log_10_ units. With a very short contact time of 1 min, a reduction in virus titer by ≈ 3.6 log_10_ units was achieved with the 0.5% SDS + 0.5% CA combination, and the 0.5% SDS + 0.5% GA combination was slightly less effective with a virus titer reduction of ≈ 2.7 log_10_ units.

**Figure 3:**
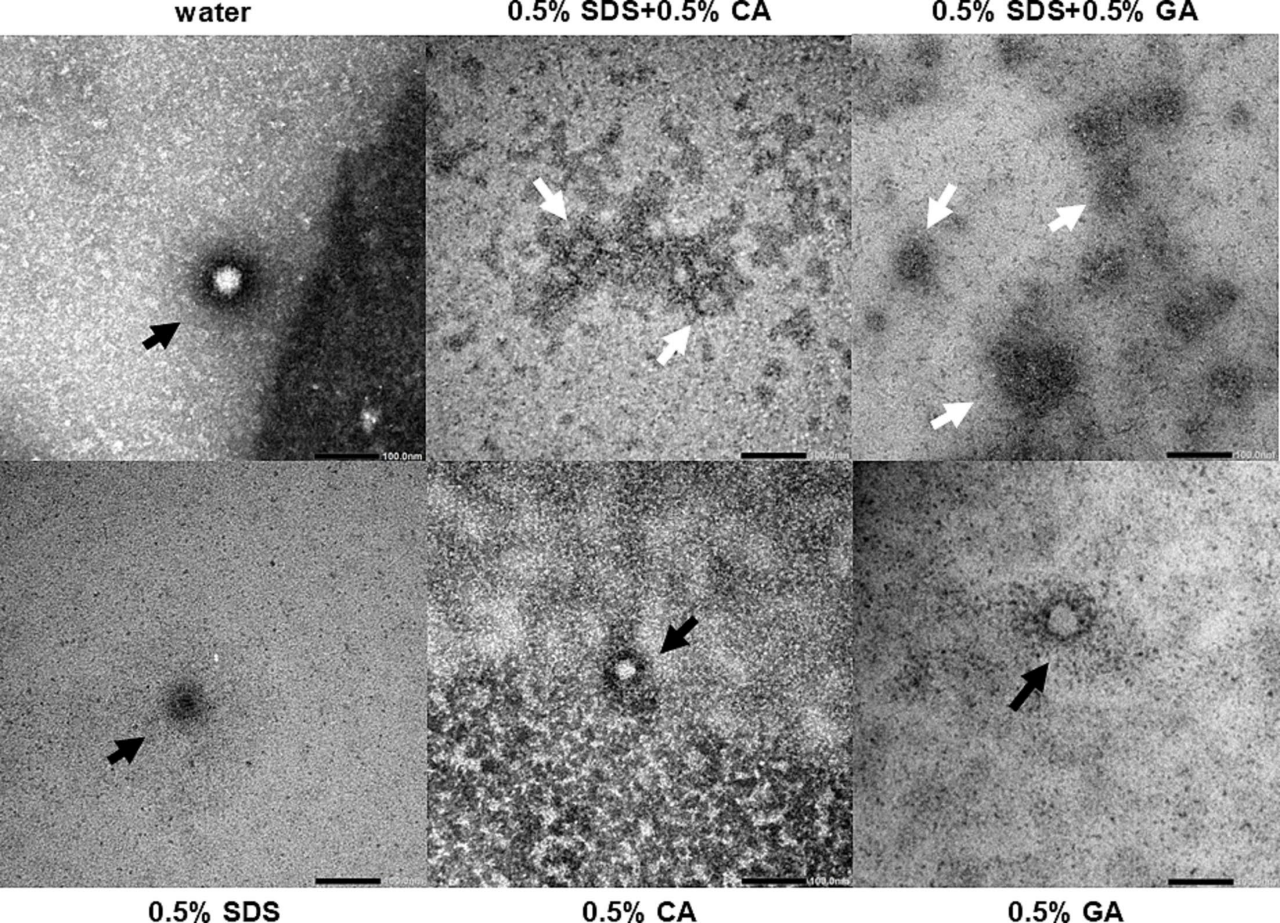
Electron micrographs of purified FCV treated with different combinations of SDS, CA, and GA. Representative images of virus particles are shown (bars = 100 nm). Black arrows indicate particles with intact capsid outlines, and white arrows indicate the remnants of a damaged virus particle.

Since the mechanism of action of SDS on non-enveloped viruses is most likely denaturation of the capsid protein, transmission electron microscopy was performed to visualize the FCV particles that had been treated with 0.5% SDS + 0.5% CA and 0.5% SDS + 0.5% GA and each of the single ingredients. Upon treatment with 0.5% SDS, CA, or GA, the capsid appeared to be intact with no gaps along the perimeter where the capsid staining is (black arrows), although the staining was altered and less intense compared to the untreated FCV (Figure 3). Upon treatment with 0.5% SDS + 0.5% CA and 0.5% SDS + 0.5% GA, the stained structures did not resemble intact capsids and appeared to be clustered in patches with undefined staining (white arrows). These findings indicate that the FCV capsids have been disrupted upon treatment by the combinations, which could not be achieved by treatment with only SDS, CA, or GA at pH 4.7, suggesting a synergistic effect of using SDS in combination with either acid.

**Figure 4:**
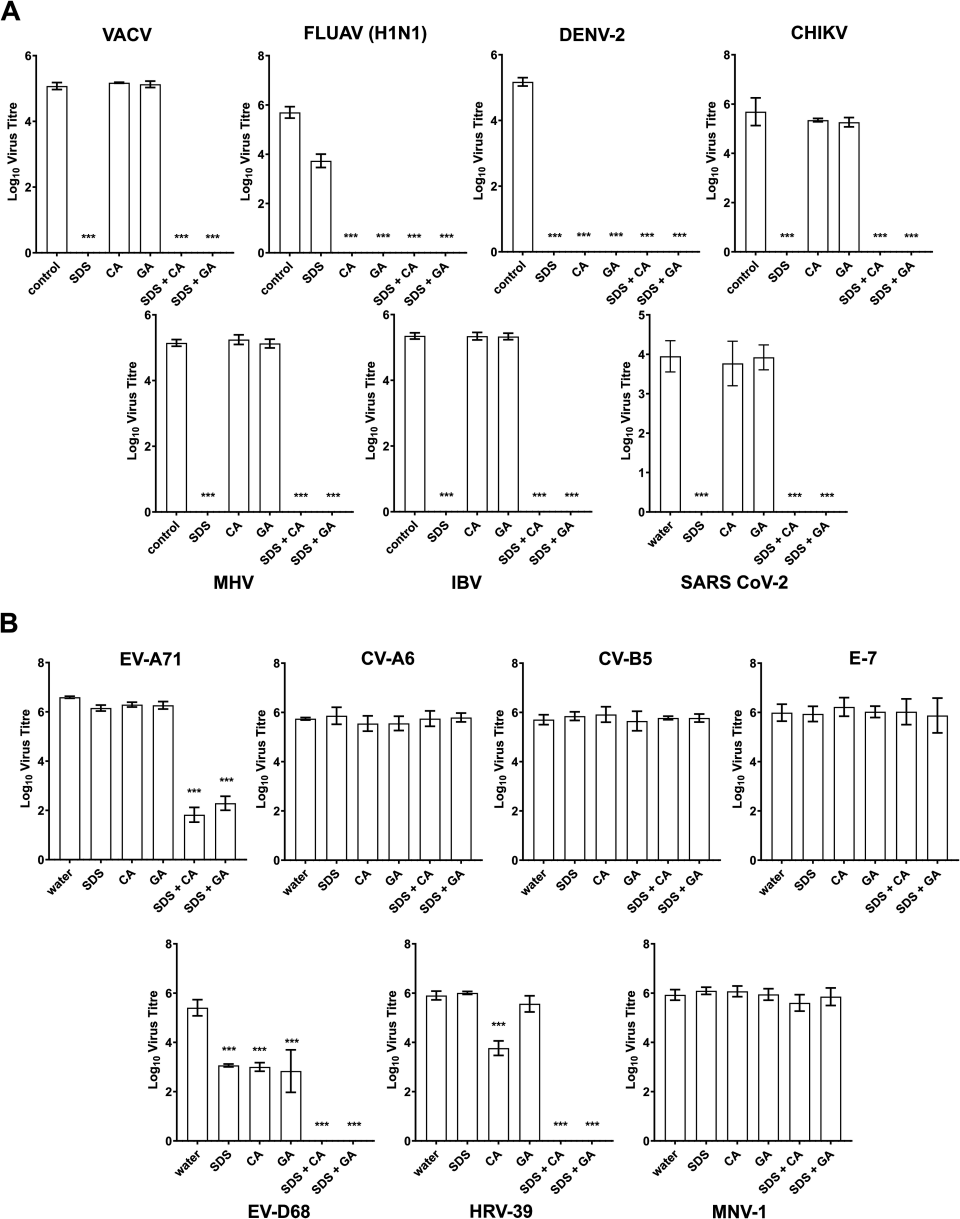
Virucidal efficacies of SDS, CA, and GA alone and in combinations against other viruses. **(A)** Virus titers of enveloped and **(B)** non-enveloped viruses after 10-min treatment with 0.5% SDS, 0.5% CA, 0.5% GA, and in combinations with SDS/CA and SDS/GA. Statistical analysis was performed using one-way ANOVA (Dunnett’s) compared against water as a control sample, ***: *p* > 0.001. The absence of titer indicated that no plaques were observed in samples with virus titers below the detection limit determined using cell viability assays (Supplementary Table S6).

The synergistic virucidal combination of SDS with CA or GA was further evaluated against various viruses of relevance to disinfectant testing as well as specific interest in Singapore, where the study is conducted, including enveloped and other non-enveloped viruses to assess their potential application as broad-spectrum virucidal agents. All the enveloped viruses evaluated were observed to be completely inactivated within 10 min (Figure 4A) when treated with 0.5% SDS + 0.5% CA (SDS + CA) and 0.5% SDS + 0.5% GA (SDS + GA). The results were expected as enveloped viruses are generally known to be sensitive to inactivation by surfactants such as SDS that solubilize the viral lipid membrane. Interestingly, dengue virus type 2 (DENV2) and influenza virus H1N1 were found to be sensitive to inactivation by CA or GA alone at pH 4.7, with no viable virus detected after 10-min treatment, implying a lower resistance compared to other enveloped viruses to even slightly acidic conditions.

For the non-enveloped viruses, human rhinovirus 39 (HRV-39) and human enterovirus D68 (EV-D68) were completely inactivated after treatment with SDS + CA and SDS + GA combinations (Figure 4B). EV-A71 was found to be highly sensitive to treatment with both combinations as virus titers were reduced close to the detection limit although live virus could still be detected after the 10-min treatment. Interestingly, HRV-39 also showed some sensitivity to 0.5% CA, and EV-D68 was also found to be sensitive to SDS, CA, and GA at 0.5%, although the single active ingredients were unable to completely inactivate the virus samples within the stipulated contact time. No virus inactivation was observed for echovirus 7 (E-7), coxsackievirus A6 (CV-A6), coxsackievirus B5 (CV-B5) and murine norovirus 1 (MNV-1). A cell viability assay was also performed on the tested samples and all cell lines were used to determine the detection limit for viral plaque assays (Supplementary Table S6).

## Discussion

Efficient inactivation of non-enveloped viruses has always required the use of harsh, reactive chemicals that are difficult to formulate into consumer products that come into direct extended contact with the human skin. Building upon existing research on SDS + acid combinations, our study explored the potential of such combinations as virus inactivation agents, and we have demonstrated that SDS, a common detergent present in many consumer products, in combination with CA and GA, can inactivate several non-enveloped viruses, FCV, EV-A71, EV-D68, and HRV-39, at slightly acidic pH 4.7. The pH for its activity is crucial, as SDS typically requires a low pH (approximately 2.5) to function effectively as a protein denaturant, which would have made it inactive in consumer products typically formulated at a pH of or above 4.5. While there have been some studies on the antimicrobial (Maktabi et al., 2018; Burel et al., 2021) or virucidal (Hong et al., 2015; Sato et al., 2020; Hu et al., 2024) properties of citric acid used alone or in combination with other chemicals, particularly at low pH, this is the first report of virus inactivation activity for glutamic acid. While both citric acid and glutamic acid in combination with SDS performed similarly with a 10-min contact time, we noted a difference in efficacy when the contact time was reduced (Figure 2), implying that glutamic acid may be the slower-acting or less effective of the two acids. More research is needed to dissect the actual mechanism of action and whether the two acids react differently to virus particles and SDS to produce the virucidal effect observed.

In addition, we have noticed stark differences in the sensitivity of viruses from the same family. Between FCV and MNV-1, comparative studies on their sensitivity to different inactivation treatments had been performed previously reporting FCV, a respiratory virus, as more sensitive to extreme pH and chlorine, while MNV, an enteric virus, was reported to be more sensitive to alcohols, even though MNV is considered to be overall more stable than FCV (Cromeans et al., 2014; Cannon et al., 2006). For the human enteroviruses, the respiratory pathogens HRV-39 and EV-D68 were found to be much more sensitive than the other viruses, which again could be attributed to the fact that their capsids do not need to be as acid-resistant as the other serotypes, which are predominantly enteric pathogens. While it appears that enteric pathogens, which are more acid-resistant, are also less sensitive to the SDS + CA and SDS + GA combinations, it does not fully explain why EV-A71 and CV-B5 behaved so differently in this study. Both are enteric pathogens with evidence of their presence in respiratory droplets (Royston and Tapparel, 2016) and sputum (Tan et al., 2023), but only EV-A71 was sensitive to both combinations and CV-B5 was completely resistant under the test conditions. Further research is necessary to unravel the actual mechanism of inactivation by the SDS + acid combinations to explain the differences in sensitivity observed, and this understanding could be beneficial in the design of new disinfectant actives in the future. Finally, as this is a pilot study demonstrating the potential of SDS + acid combinations being applied to the development of consumer products, the research is still in its early phase, and rigorous formulation optimization and *in vivo* testing are needed to assess its true efficacy and cytotoxicity in humans specific to the route of application.

## Data Availability

The original contributions presented in the study are included in the article/Supplementary material, further inquiries can be directed to the corresponding author.
