# Supplementary material for: Non-enveloped virus inactivation potency of sodium dodecyl sulfate with citric and glutamic acids
Source: Front Microbiol. 2025 Feb 19;16:1535404. doi: 10.3389/fmicb.2025.1535404 (PMC11880254; doi:10.3389/fmicb.2025.1535404)
Supplement: Supplementary file 1 [file Data_Sheet_1.pdf]

Non-enveloped virus inactivation potency of sodium dodecyl sulphate with citric and glutamic acids

Yong Wah Tan[1], Chwee Fern Bok[1], Brenda Jun Fang Lim[1], Li Ying Kong[2], Kian Sim Goo[2], Yoshiki Ishida[2], Jiquan Liu[2], Chun Song Chua[2], Justin Jang Hann Chu[1,3,4,5*]

[1]Institute of Molecular and Cell Biology (IMCB), Agency for Science, Technology and Research (A*STAR), 61 Biopolis Drive Singapore 138673, Singapore.

[2]Singapore Innovation Center, The Procter & Gamble Company, Singapore, Singapore

[3]Biosafety Level 3 Core Facility, Yong Loo Lin School of Medicine, National University of Singapore, Singapore, Singapore

[4]Laboratory of Molecular RNA Virology and Antiviral Strategies, Department of Microbiology and Immunology, Yong Loo Lin School of Medicine, National University of Singapore, Singapore.

[5]Infectious Disease Translation Research Programme, Yong Loo Lin School of Medicine, National University of Singapore, Singapore, Singapore

*** Correspondence:**
Justin Jang Hann Chu
miccjh@nus.edu.sg

Supplementary Information

Supplementary Table S1 Cell lines and culture media used for propagation of viruses. EMEM: Eagle's minimum essential medium, DMEM: Dulbecco's modified Eagle medium, RPMI: RPMI-1640, FBS: fetal bovine serum, A549-ACE2: A549 cell line stably expressing human angiotensin converting enzyme 2 (ACE2).

Virus	Cell Line	Media
FCV	Crandell-Rees Feline Kidney Cell (CRFK) (ATCC #CCL-94)	EMEM + 2% FBS
MNV-1	RAW264.7 (ATCC #TIB-71)	EMEM + 2% FBS
VACV, CV-B5, EV-D68	Vero (ATCC #CCL-81)	DMEM + 2% FBS
HRV-39	H1-HeLa (ATCC #CRL-1958)	EMEM + 2% FBS
FLUAV H1N1	Madin-Darby canine kidney (MDCK) (ATCC #NBL-2)	EMEM
EV-A71, CV-A6, E-7	Rhabdomyosarcoma (RD) (ATCC #CCL-136)	DMEM + 2% FBS
CHIKV, DENV-2	Baby hamster kidney (BHK)	RPMI + 2% FBS
IBV	H1299	DMEM + 2% FBS
MHV	H2.35	DMEM + 2% FBS
SARS CoV-2	A549-ACE2	DMEM + 2% FBS

Supplementary Table S2 Plaque assay overlay medium composition and incubation temperatures for different viruses. EMEM: Eagle's minimum essential medium, DMEM: Dulbecco's modified Eagle medium, RPMI: RPMI-1640, FBS: fetal bovine serum, CMC: carboxymethylcellulose, TPCK-Tosyl phenylalanyl chloromethyl ketone, MCC: microcrystalline cellulose.

**Virus**	**Overlay Media**	**Temperature (°C)**
FCV	EMEM + 0.8% CMC + 2% FBS	35
MNV-1	EMEM + 1% CMC + 2% FBS	37
HRV39, EV-D68	DMEM + 0.5% agarose + 2% FBS	33
EV-A71, CV-A6, E-7	DMEM + 0.5% agarose + 2% FBS	37
CV-B5	DMEM + 0.5% agarose + 2% FBS	37
VACV	EMEM + 1.5% CMC + 2% FBS	37
FLUAV (H1N1)	EMEM + 0.3% agarose + 4μg/ml trypsin (TPCK treated)	37
DENV-2	RPMI + 1% CMC + 2% FBS	37
CHIKV	RPMI + 1% CMC + 2% FBS	37
MHV	DMEM + 1.2% CMC + 2% FBS	35
IBV	DMEM + 0.5% agarose + 2% FBS	37
SARS CoV-2	DMEM + 1.2% MCC + 2% FBS	37

Supplementary Table S3 Cell viability assay performed on treatment samples without virus in CRFK cells at different pH. Cytotoxicity is observed when viability drops below 80% (values marked in red). The minimum concentration for plaque assay detection is 100 PFU/ml all samples in all cell lines without toxicity. Toxicity observed in dilution factor 10^-1 wells increases the minimum detection concentration to 1000 PFU/ml.

		Dilution factor (1/10^χ)			
**Actives**	**pH**	**-1**	**-2**	**-3**	**-4**
5% SDS	2.5	-27.50	100.45	105.35	105.69
5% SDS + 1% CA	2.5	-31.12	93.96	95.70	100.03
5% SDS + 1% GA	2.5	-31.39	96.96	96.00	93.82
5% SDS	4.7	-26.77	105.47	103.90	100.06
5% SDS + 1% CA	4.7	-17.49	104.53	104.56	102.21
5% SDS + 1% GA	4.7	-23.68	102.74	97.87	96.95
5% SDS	7	-23.72	104.59	97.72	94.96
5% SDS + 1% CA	7	-18.07	110.95	104.38	104.14
5% SDS + 1% GA	7	-23.21	104.83	101.75	98.45
5% SDS	10	-23.19	102.36	99.22	97.13
5% SDS + 1% CA	10	-23.19	102.36	99.22	97.13
5% SDS + 1% GA	10	-18.15	104.59	103.46	106.69

Supplementary Table S4 Cell viability assay performed on treatment samples without virus in CRFK cells at pH 4.7. Cytotoxicity is observed when viability drops below 80% (values marked in red). The minimum concentration for plaque assay detection is 100 PFU/ml all samples in all cell lines without toxicity. Toxicity observed in dilution factor 10^-1 wells increases the minimum detection concentration to 1000 PFU/ml.

		**Dilution factor (1/10^χ)**			
		**-1**	**-2**	**-3**	**-4**
SDS (%)					
2.5		5.55	101.16	99.72	98.41
1		96.7	108.6	109.1	110.5
0.5		109.2	114.4	110.5	100.6
0.2		109.9	106.9	111.4	116.8
Combinations					
SDS (%)	CA (%)				
2.5	1	-1.07	102.74	104.32	94.31
1	1	106.71	105.37	101.25	98.68
0.5	1	109.31	103.37	100.87	100.15
0.2	1	103.49	101.60	103.25	97.82
0.5	0.5	110.30	105.24	101.10	101.95
0.5	0.2	101.51	110.28	104.05	96.36
0.5	0.1	108.94	107.33	103.80	100.81
SDS (%)	GA (%)				
2.5	1	-1.80	103.38	106.58	97.28
1	1	102.14	105.76	99.35	91.01
0.5	1	101.88	103.26	99.24	94.63
0.2	1	109.17	104.11	100.41	101.49
0.5	0.5	102.80	110.23	102.38	96.33
0.5	0.2	107.18	103.39	100.34	101.74
0.5	0.1	108.26	115.89	107.42	98.17

Supplementary Table S5 Statistical analysis for data presented in Figure 1A. Analysis was performed with separate one-way ANOVA with Tukey's multiple comparisons test for each sample type (SDS, SDS/CA or SDS/GA) at different concentrations including water (negative control). ns: not significant.

**Tukey's multiple comparisons test**	**Significance**	***p***
SDS only		
water (negative control) vs. 2.5% SDS	ns	0.92
water (negative control) vs. 1% SDS	ns	0.7684
water (negative control) vs. 0.5% SDS	ns	0.8355
water (negative control) vs. 0.2% SDS	ns	0.0821
2.5% SDS vs. 1% SDS	ns	0.9961
2.5% SDS vs. 0.5% SDS	ns	0.4011
2.5% SDS vs. 0.2% SDS	*	0.0232
1% SDS vs. 0.5% SDS	ns	0.2545
1% SDS vs. 0.2% SDS	*	0.0135
0.5% SDS vs. 0.2% SDS	ns	0.3584
SDS/CA		
water (negative control) vs. 2.5% SDS/1% CA	***	<0.001
water (negative control) vs. 1% SDS/1% CA	***	<0.001
water (negative control) vs. 0.5% SDS/1% CA	***	<0.001
water (negative control) vs. 0.2% SDS/1% CA	ns	0.9745
2.5% SDS/1% CA vs. 1% SDS/1% CA	***	<0.001
2.5% SDS/1% CA vs. 0.5% SDS/1% CA	***	<0.001
2.5% SDS/1% CA vs. 0.2% SDS/1% CA	***	<0.001
1% SDS/1% CA vs. 0.5% SDS/1% CA	ns	0.8007
1% SDS/1% CA vs. 0.2% SDS/1% CA	***	<0.001
0.5% SDS/1% CA vs. 0.2% SDS/1% CA	***	<0.001
SDS/GA		
water (negative control) vs. 2.5% SDS/1% GA	***	<0.001
water (negative control) vs. 1% SDS/1% GA	***	<0.001
water (negative control) vs. 0.5% SDS/1% GA	***	<0.001
water (negative control) vs. 0.2% SDS/1% GA	ns	0.2339
2.5% SDS/1% GA vs. 1% SDS/1% GA	***	<0.001
2.5% SDS/1% GA vs. 0.5% SDS/1% GA	***	<0.001
2.5% SDS/1% GA vs. 0.2% SDS/1% GA	***	<0.001
1% SDS/1% GA vs. 0.5% SDS/1% GA	ns	0.9596
1% SDS/1% GA vs. 0.2% SDS/1% GA	***	<0.001
0.5% SDS/1% GA vs. 0.2% SDS/1% GA	***	<0.001

Supplementary Table S6 Cell viability assay performed on treatment samples without virus in multiple cell lines. Cytotoxicity is observed when viability drops below 80% (values marked in red). The minimum concentration for plaque assay detection is 100 PFU/ml all samples in all cell lines without toxicity. Toxicity observed in dilution factor 10^-1 wells increases the minimum detection concentration to 1000 PFU/ml.

	**Dilution factor (1/10^χ)**	**H1299**	**H1-HeLa**	**H2.35**	**RD**	**BHK**	**MDCK**	**RAW264.7**	**Vero**	**A549-ACE2**
0.5% SDS	**-1**	60.32	103.71	79.94	54.08	62.85	125.82	99.08	105.96	97.77
	**-2**	108.83	102.75	103.57	106.59	95.97	128.22	96.28	103.81	119.59
	**-3**	105.72	100.75	99.91	112.19	97.93	116.15	90.57	103.40	104.85
	**-4**	103.94	101.77	97.74	107.10	97.59	119.75	94.43	102.73	108.2196
0.5% CA	**-1**	57.62	105.70	81.62	58.32	91.50	117.27	100.67	104.39	110.67
	**-2**	105.96	102.25	103.43	106.81	98.04	124.30	98.98	99.07	104.32
	**-3**	101.38	101.71	98.08	106.91	98.14	112.40	95.91	96.75	98.58
	**-4**	99.02	103.16	95.52	101.96	96.72	107.65	98.13	95.94	97.97
0.5% GA	**-1**	64.14	106.45	81.67	55.47	88.03	120.10	104.18	101.99	88.67
	**-2**	108.71	102.00	102.49	101.11	92.25	133.47	105.89	95.39	118.84
	**-3**	105.82	102.00	95.24	99.88	91.42	104.50	95.57	94.72	99.63
	**-4**	103.36	102.47	95.24	99.30	90.46	100.02	98.42	96.85	93.79
0.5% SDS + 0.5% CA	**-1**	56.78	102.65	84.72	46.00	87.31	138.93	100.64	103.52	127.47
	**-2**	96.64	102.58	105.75	103.35	100.27	137.85	101.91	102.62	132.37
	**-3**	97.85	100.65	100.76	104.45	98.02	132.23	96.05	101.54	107.55
	**-4**	95.63	100.81	100.29	101.21	96.98	126.16	100.45	101.75	91.70
0.5% SDS + 0.5% GA	**-1**	67.40	101.83	80.33	59.83	70.78	141.44	98.69	109.06	118.89
	**-2**	111.51	104.92	105.98	105.46	97.76	134.43	101.46	105.54	119.48
	**-3**	108.16	101.33	101.02	113.42	99.75	127.87	95.79	104.95	123.53
	**-4**	105.17	100.98	99.19	107.90	99.92	120.97	96.50	104.41	124.21
